# 507 Earlier Graft Takedown Using Autologous Keratinocyte Suspension in Conjunction with Split Thickness Skin Grafting for the Treatment of Deep 2nd and 3rd Degree Burns

**DOI:** 10.1093/jbcr/irad045.104

**Published:** 2023-05-15

**Authors:** Roald Credo, Elizabeth Brown, Cris Dhaiti, Alan Pang, Akshay Raghuram, Deepak Bharadia, John Griswold

**Affiliations:** TTUHSC School of Medicine, Lubbock, Texas; Texas Tech University Health Sciences Center School of Medicine, Lubbock, Texas; TTUHSC School of Medicine, Lubbock, Texas; Texas Tech University Health Sciences Center, Lubbock, Texas; Texas Tech University Health Sciences Center, Lubbock, Texas; Texas Tech University Health Sciences Center, Lubbock, Texas; Texas Tech University Health Sciences Center, Lubbock, Texas; TTUHSC School of Medicine, Lubbock, Texas; Texas Tech University Health Sciences Center School of Medicine, Lubbock, Texas; TTUHSC School of Medicine, Lubbock, Texas; Texas Tech University Health Sciences Center, Lubbock, Texas; Texas Tech University Health Sciences Center, Lubbock, Texas; Texas Tech University Health Sciences Center, Lubbock, Texas; Texas Tech University Health Sciences Center, Lubbock, Texas; TTUHSC School of Medicine, Lubbock, Texas; Texas Tech University Health Sciences Center School of Medicine, Lubbock, Texas; TTUHSC School of Medicine, Lubbock, Texas; Texas Tech University Health Sciences Center, Lubbock, Texas; Texas Tech University Health Sciences Center, Lubbock, Texas; Texas Tech University Health Sciences Center, Lubbock, Texas; Texas Tech University Health Sciences Center, Lubbock, Texas; TTUHSC School of Medicine, Lubbock, Texas; Texas Tech University Health Sciences Center School of Medicine, Lubbock, Texas; TTUHSC School of Medicine, Lubbock, Texas; Texas Tech University Health Sciences Center, Lubbock, Texas; Texas Tech University Health Sciences Center, Lubbock, Texas; Texas Tech University Health Sciences Center, Lubbock, Texas; Texas Tech University Health Sciences Center, Lubbock, Texas; TTUHSC School of Medicine, Lubbock, Texas; Texas Tech University Health Sciences Center School of Medicine, Lubbock, Texas; TTUHSC School of Medicine, Lubbock, Texas; Texas Tech University Health Sciences Center, Lubbock, Texas; Texas Tech University Health Sciences Center, Lubbock, Texas; Texas Tech University Health Sciences Center, Lubbock, Texas; Texas Tech University Health Sciences Center, Lubbock, Texas; TTUHSC School of Medicine, Lubbock, Texas; Texas Tech University Health Sciences Center School of Medicine, Lubbock, Texas; TTUHSC School of Medicine, Lubbock, Texas; Texas Tech University Health Sciences Center, Lubbock, Texas; Texas Tech University Health Sciences Center, Lubbock, Texas; Texas Tech University Health Sciences Center, Lubbock, Texas; Texas Tech University Health Sciences Center, Lubbock, Texas; TTUHSC School of Medicine, Lubbock, Texas; Texas Tech University Health Sciences Center School of Medicine, Lubbock, Texas; TTUHSC School of Medicine, Lubbock, Texas; Texas Tech University Health Sciences Center, Lubbock, Texas; Texas Tech University Health Sciences Center, Lubbock, Texas; Texas Tech University Health Sciences Center, Lubbock, Texas; Texas Tech University Health Sciences Center, Lubbock, Texas

## Abstract

**Introduction:**

Autologous split-thickness skin grafts (STSG) have been the gold standard concerning treatment of both full and partial thickness burns. However, new treatments are emerging such as spray-on autologous keratinocyte suspensions (AKS). AKS is composed of a patient’s own keratinocytes, fibroblasts, and melanocytes. STSG in conjunction with AKS has showed promising results for the treatment of deep partial and full thickness burns. The standard graft takedown with STSG has historically been on postoperative day (POD) 6. Once graft take down occurs, occupational therapy can begin along with cessation of continuous splinting leading to more convenience and comfortability for the patient. With AKS, we propose that faster wound healing allows graft take down to occur earlier on POD 4.

**Methods:**

A retrospective chart review was conducted at TTUHSC’s UMC Burn Center looking at patients that underwent STSG with AKS for the treatment of their burn. Patients were divided based on graft takedown on POD 4 or 6. Factors such as time to complete wound closure and postoperative complications were analyzed.

**Results:**

The average age and total burn surface area in both groups was 43 years old and 28%. In the POD 4 group (n=10) the average time to healing was 2 months while the POD 6 group (n=21) had an average healing time of 3.1 months. Graft take and percentage of patients with changes in postoperative mobility and hypertrophic scarring were the same across both groups (98%, 30%, and 50%, respectively).

**Conclusions:**

While time to healing was slightly different, there was not a statistically significant difference between the two groups. From this study we can conclude that graft take down can occur earlier in patients treated with STSG in conjunction with AKS. This allows for earlier therapeutic intervention and more convenience for the patient.

**Applicability of Research to Practice:**

• Graft takedown can occur earlier on postoperative day 4 instead of 6 in patients treated with split-thickness skin grafting in conjunction with autologous keratinocyte suspension.

• Earlier graft takedown allows for earlier occupational therapy and reduces the need for extensive splinting which can be uncomfortable for the patient.

• Graft takedown can occur earlier in those treated with split-thickness skin grafting in conjunction with autologous keratinocyte suspension due to the rapid healing the suspension offers.

